# Effects of *Pelargonium sidoides* and Coptis Rhizoma 2 : 1 Mixed Formula (PS + CR) on Ovalbumin-Induced Asthma in Mice

**DOI:** 10.1155/2020/9135637

**Published:** 2020-02-28

**Authors:** Byung Gu Min, Sang Mi Park, Youn Woong Choi, Sae Kwang Ku, Il Je Cho, Young Woo Kim, Sung Hui Byun, Chung A Park, Sook Jahr Park, MinKyun Na, Sang Chan Kim

**Affiliations:** ^1^College of Pharmacy, Chungnam National University, Daejeon 34134, Republic of Korea; ^2^Korea United Pharm. Inc., Seoul 06116, Republic of Korea; ^3^College of Korean Medicine, Daegu Haany University, Gyeongsan 38610, Republic of Korea; ^4^School of Korean Medicine, Dongguk University, Gyeongju 38066, Republic of Korea; ^5^Department of Pharmaceutical Engineering, Daegu Haany University, Gyeongsan 38610, Republic of Korea

## Abstract

*Pelargonium sidoides* (PS) is traditionally used to treat respiratory and gastrointestinal infections, dysmenorrhea, and hepatic disorders in South Africa. Coptis Rhizoma (CR) is used to treat gastroenteric disorders, cardiovascular diseases, and cancer in East Asia. In the present study, we intended to observe the possible beneficial antiasthma effects of PS and CR on the ovalbumin- (OVA-) induced asthma C57BL/6J mice. Asthma in mice was induced by OVA sensitization and subsequent boosting. PS + CR (300 and 1,000 mg/kg; PO) or dexamethasone (IP) was administered once a day for 16 days. The changes in the body weight and gains, lung weights and gross inspections, total and differential cell counts of leukocytes in bronchoalveolar lavage fluid (BALF), serum OVA-specific immunoglobulin E (OVA-sIgE) levels, interleukin-4 (IL-4) and IL-5 levels in BALF and lung tissue homogenate, and IL-4 and IL-5 mRNA levels in lung tissue homogenates were analyzed with lung histopathology: mean alveolar surface area (ASA), alveolar septal thickness, numbers of inflammatory cells, mast cells, and eosinophils infiltrated in the alveolar regions, respectively. Significant increases in lung weights, total and differential cell counts of leukocytes in BALF, serum OVA-sIgE levels, and IL-4 and IL-5 levels in BALF and lung tissue homogenate were observed in OVA control as compared to those of intact control. In addition, OVA control showed a significant decrease in mean ASA and increases in alveolar septal thickness, numbers of inflammatory cells, mast cells, and eosinophils infiltrated in alveolar regions. However, these allergic and inflammatory asthmatic changes were significantly inhibited by PS + CR in a dose-dependent manner. In this study, PS + CR showed dose-dependent beneficial effects on OVA-induced asthma in mice through anti-inflammatory and antiallergic activities. Therefore, it is expected that PS + CR have enough potential as a new therapeutic agent or as an ingredient of a medicinal agent for various allergic and inflammatory respiratory diseases including asthma.

## 1. Introduction

Asthma is a chronic inflammatory disease of the lungs, characterized by airway hyperresponsiveness to both inhaled allergens and nonspecific stimuli [1]. The airway hyperresponsiveness results from epithelial injury caused by the accumulation of activated eosinophils and mast cells within the respiratory tract [2]. There is also convincing evidence that increased immunoglobulin E (IgE) levels and goblet-cell hyperplasia are observed in asthma [3]. The prevalence of asthma is increasing worldwide, and it has become a significant cause of health challenge especially in developed countries [4].

Type 2 helper *T* (Th2) cells seem to play a pivotal role in immune dysfunction, which contributes to the development of asthma [5–7]. In addition, it has been observed that the Th2-associated cytokines (e.g., interleukin-4 (IL-4), IL-5, and IL-13) were released by the airway epithelial cells. IL-4 has important roles in allergic inflammation and airway remodeling [8] and promotes the differentiation of B-lymphocytes, which lead to IgE generation [9]. IL-5 is the most specific to eosinophils and is responsible for eosinophil growth, differentiation, mobilization, activation, recruitment, and survival [10]. Eosinophils differentiate within tissues undergoing an allergic response, including asthma [11]. Thus, regulating the IL-4 or IL-5 is a useful therapeutic approach in allergic asthma [4, 12].

*Pelargonium sidoides* (PS), the important traditional medicine for treatment of diarrhea and dysentery in South Africa, has antibacterial, antifungal, antimycobacterial, and immunomodulatory properties [13]. Moreover, PS can prevent asthma attacks during upper respiratory tract viral infections and reduce rhinovirus infection by modulating viral binding proteins [14, 15]. Coptis Rhizoma (CR) is used to treat of bacillary dysentery, typhoid, tuberculosis, epidemic cerebrospinal meningitis, pertussis, and other diseases [16].

Recent studies have shown that these herbs have antioxidant, anticancer, and anti-inflammatory pharmacological activities [17, 18]. In addition, we previously reported that PS + CR had potent anti-inflammatory activities, *in vitro* and *in vivo* [19].

Ovalbumin- (OVA-) induced asthma C57BL/6J mice are a representative asthma animal model resembling human asthma [20, 21], and dexamethasone (DEXA) is a well-known glucocorticoid and is widely used as an anti-inflammatory control drug for development of new antiasthmatic agents [20, 22].

In this study, we intended to observe the possible beneficial antiasthma effects of PS + CR (2 : 1 mixed formula, w:w) on OVA-induced asthma C57BL/6J mice, our hope being to help develop a potent alternative antiasthmatic natural agent or functional food.

## 2. Materials and Methods

### 2.1. Chemicals and Reagents

Carboxymethyl cellulose (CMC) sodium salt, DEXA-water soluble, OVA, trypan blue, aluminum hydroxide gel, and Al(OH)_3_ were obtained from Sigma-Aldrich (St. Louis, MO, USA). Isoflurane was obtained from Hana Pharm. Co. (Hwaseong, Korea). IL-4, IL-5, and an OVA-specific IgE (OVA-sIgE) ELISA kit were obtained from MyBioSource (San Diego, CA, USA). TRIzol reagent was obtained from Invitrogen (Carlsbad, CA, USA).

### 2.2. Preparations of PS + CR

PS and CR were prepared and supplied by Korea United Pharm. Inc. (Seoul, Korea) [19]. In this study, CMC stock solution was dissolved in distilled water at a concentration of 5 mg/ml and autoclaved at 121°C for 20 min using a general and commercial autoclave (DAC-40, Woori Science, Seoul, Korea). Appropriate amounts of herbal mixture were directly suspended in 0.5% CMC solution. Specifically, PS : CR in ratios of 200 mg:100 mg (300 mg) and 666.67 mg:333.33 mg (1000 mg) were dissolved in 10 ml of 0.5% CMC solutions.

### 2.3. Animal Treatment

**Figure 1:**
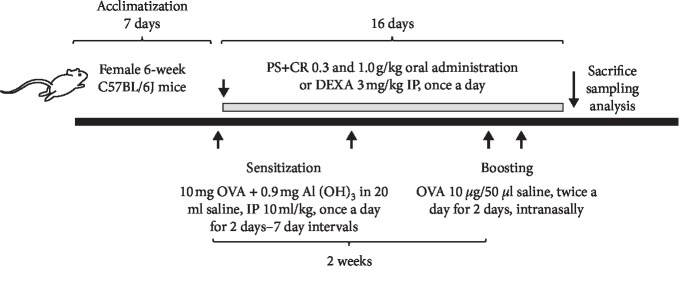
Experimental design used in this study. Analysis was as follows: body weight and gains, lung weights, total and differential cell counts of leukocytes in BALF, serum OVA-sIgE levels, IL-4 and IL-5 levels in BALF and lung tissue homogenate, IL-4 and IL-5 mRNA levels in lung tissue homogenate, and lung histopathology.

C57BL/6J mice (six weeks old, weighting 16–18 g) were purchased from Dae Han Bio Link (Eumseong, Korea) and used after acclimatization for seven days. Animals were allocated at five for each polycarbonate cage in a temperature (20°C–25°C) and humidity (50–55%) controlled room. The light:dark cycle was 12 : 12 h, and standard rodent chow (Purinafeed, Seongnam, Korea) and tap water were supplied *ad libitum*. All laboratory animals were treated according to the international regulations of the usage and welfare of laboratory animals, as approved by the Institutional Animal Care and Use Committee (Approval no. DHU2018-050) in Daegu Haany University (Gyeongbuk, Korea) prior to the animal experiment. Ten mice in each group, in a total of five groups, were selected based on the body weight deviations (intact control, average 18.16 ± 0.56 g, range 17.3–17.3 g; OVA groups, average 18.18 ± 0.61 g, range 17.1–19.2 g) (Figure 1). PS + CR was orally administered in a volume of 10 ml/kg (as equivalent to 300 and 1,000 mg/kg in PS + CR) once a day for 16 days, using a zonde attached to a 1 ml syringe. The dosage of PS + CR was selected based on our previous *in vivo* anti-inflammatory efficacy test [19]. DEXA was prepared by dissolving in saline as a 0.3 mg/ml concentration and used as a reference drug. DEXA was intraperitoneally (IP) injected at a volume of 10 ml/kg (as equivalent to 3 mg/kg) once a day for 16 days. To provide the same stresses caused by administration, equal volumes of 0.5% CMC solutions were orally administered to intact and OVA control mice. PS + CR or DEXA was given an hour after the end of OVA sensitization and boosting. Actual compositions of test materials and vehicles are listed in Table 1.

### 2.4. Mouse Sensitization and Challenge with OVA

Asthma in C57BL/6J mice was induced by OVA sensitization (IP, 10 mg OVA absorbed to 0.9 mg Al(OH)_3_ containing 20 ml saline, once a day for two days, at seven day intervals) and boosting (intranasal treatment of OVA 10 *μ*g/50 *μ*l saline, twice a day for two continuous days) according to previous OVA-induced asthma mouse experiments [20, 22]. One week after the first OVA sensitization, the mice received a second OVA solution. At seven days after the second OVA sensitization, the mice were intranasally challenged with OVA (10 *μ*g/50 *μ*l) twice a day for 2 continuous days using yellow tips attached to micropipettes.

### 2.5. Measurement of Body Weights

Changes of body weights were measured one day before initial herbal mixture administration, on the day of the first herbal mixture administration, and at 1, 7, 14, 15, and 16 days after initial DEXA or herbal mixture administration using an automatic electronic balance (XB320 M, Precisa Instruments, Dietikon, Switzerland). To reduce the individual differences, the body weight gains during the 16 days of the experimental periods were calculated as body weight at 24 h after the last (16^th^) administration minus body weight on the day of first administration.

### 2.6. Gross Inspection and Measurement of Lung Weights

At 24 h after the last administration and 25 h after the second OVA boosting, we separated individual lungs that were under anesthesia with 2 to 3% isoflurane in the mixture of 70% N_2_O and 28.5% O_2_ using rodent inhalation anesthesia apparatus (Surgivet, Waukesha, WI, USA) and rodent ventilator (Model 687, Harvard Apparatus, Cambridge, UK) and performed the gross inspections with imaging by a commercial digital camera (FinePix S700, Fujifilm, Tokyo, Japan). After that, the weights of individual lung were measured at Gram levels (absolute wet weights) using an automatic electronic balance (XB320 M, Precisa Instruments, Dietikon, Switzerland). Relative lung weights were expressed as percentage of body weights [(absolute lung wet-weights/body weight at sacrifice) × 100].

### 2.7. Lung Sampling

After lung weight measurement, two 3–0 nylon ligations were conducted on the left secondary bronchus and right lower secondary bronchus. We used the left lung for histopathological inspections, the right lung upper and middle lobes for BALF collection, and the right lung lower lobes for cytokine analysis.

### 2.8. BALF Collection

After 3–0 nylon ligations, 1 ml of physiological saline was instilled in the tracheal cannula (20 G) with subsequent aspiration with a syringe. This was repeated twice, yielding two samples from each animal according to previous methods [20, 22] with some modification. The total number of cells was counted by an automated cell counter (Countess C10281; Invitrogen) under trypan blue stain. In addition, total leukocyte numbers and differential counts, lymphocytes, neutrophils, eosinophils, and monocytes, were detected by an automated hematology cell counter (Cell-DYN3700, Abbott Laboratories, Abbott Park, IL, USA).

### 2.9. Preparation of Lung Tissue Homogenates

Lung tissues (right lower lobes) were homogenized using a bead beater (TacoTMPre, GeneResearch Biotechnology Corp., Taichung, Taiwan) and ultrasonic cell disruptor (KS-750, Madell Technology Corp., Ontario, CA, USA) in an equal volume of normal saline.

### 2.10. Serum OVA-sIgE and Cytokines Measurement

Blood was collected through the vena cava of mice under inhalation anesthesia at sacrifice 25 h after the 16th administration. Serum samples were obtained by centrifugation and stored at −150°C in an ultradeep freezer (MDF-1156, Sanyo, Tokyo, Japan) until analysis. The serum was diluted 1 : 25 in assay diluent buffer, and OVA-sIgE was measured using a mouse OVA-sIgE ELISA kit, according to the manufacturer's protocol. Optical density was measured at 450 nm by a microplate reader (Sunrise, Tecan, Männedorf, Switzerland). Lung and BALF IL-4 and IL-5 levels were also measured using mouse IL-4 and IL-5 ELISA kits at 450 nm, respectively.

### 2.11. Quantitative RT-PCR Analysis

RNA was extracted using TRIzol reagent from prepared individual lung tissue homogenate according to the method described in a previous study [23]. The RNA concentrations and quality were measured by a Real-Time System (CFX96 TM, Bio-Rad, Hercules, CA, USA). To remove contaminating DNA, samples were treated with recombinant DNase I (DNA-free DNA Removal kit; Ambion; Thermo Fisher Scientific, Inc., Waltham, MA, USA). RNA was reverse-transcribed using the High-Capacity cDNA Reverse Transcription kit (Applied Biosystems; Thermo Fisher Scientific, Inc.) following the manufacturer's instructions. The PCR cycling conditions were as follows: initial predenaturation of 95°C for 1 min, denaturation for 15 sec, annealing at 55°C–65°C for 20 sec, and extension of 72°C for 30 sec. A total of 50 cycles were performed. The glyceraldehyde 3-phosphate dehydrogenase (GAPDH) RNA was used as a control for tissue integrity in all samples. PCR primer sequences are listed in Table 2. For quantitative analysis, the intact control lung tissue was used as the control, and the relative expressions of IL-4 and IL-5 were calculated using the 2^–ΔΔCq^ method [24].

### 2.12. Histopathology

Approximately equal regions of the left lateral lobe of an individual lung were cross-trimmed according to previously established methods [25]. Representative sections were stained with hematoxylin and eosin (H&E) for general histopathology, Congo red for eosinophils, or toluidine blue for mast cells, according to previously established methods [20, 26, 27]. After that, the histological profiles of individual cross-trimmed left lateral lobes of lung tissue were observed by using a light microscope (Model Eclipse 80*i*, Nikon, Tokyo, Japan) while being blinded to group distribution. To observe more details of any changes, the mean ASA (%/mm^2^) reflected the pulmonary functions, gas exchange capacities [20, 28], and mean alveolar septal thickness (*μ*m) and numbers of inflammatory cells, eosinophils, and mast cells infiltrated in the alveolar regions (cells/mm^2^) [20, 21, 27] were calculated for histomorphometrical analysis using a computer-assisted image analysis program (*i*Solution FL ver 9.1, IMT *i*-Solution Inc., Vancouver, Quebec, Canada).

### 2.13. Statistical Analysis

Values are expressed as mean ± SD of ten mice. One-way ANOVA was used to assess the multiple comparison tests for different dose groups, followed by Levene's test or a least-significant differences multicomparison test [24, 29, 30]. Differences were considered significant at *p* < 0.05. In addition, the percent changes between intact vehicle and OVA control mice were calculated to monitor the severities of asthmatic changes induced by OVA sensitization and boosting, and the percent changes between OVA control mice and PS + CR 300 and 1,000 mg/kg or DEXA 3 mg/kg treated mice were also calculated to help us understand the efficacy, according to our previous reports [31, 32] as follows:

- Percent changes as compared with intact control (%) = ((data of OVA control−data of intact control)/data of intact control) × 100
- Percent changes as compared with OVA control (%) = ((data of PS + CR or DEXA treated mice−data of OVA control)/data of OVA control) × 100

## 3. Results

### 3.1. Changes in the Body Weights

**Figure 2:**
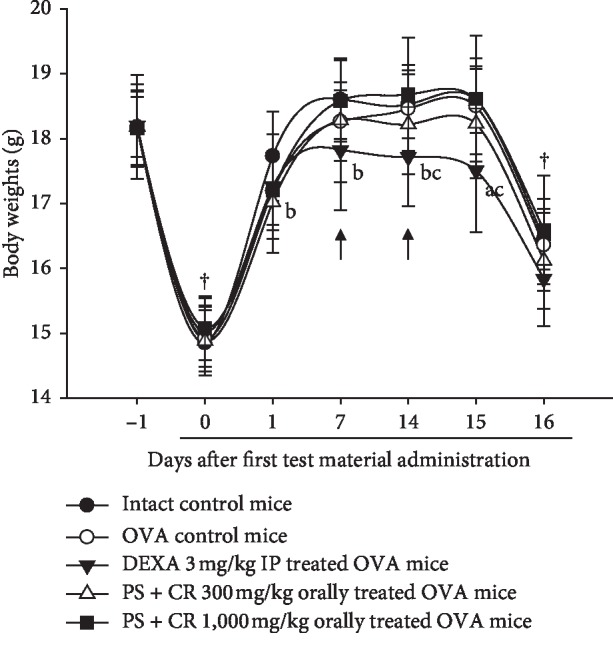
Body weights changes in intact or OVA-induced asthmatic mice. No significant changes in the body weights were detected in OVA control as compared to those of intact control mice throughout the experimental period. In addition, no significant changes in the body weights were detected in PS + CR 300 or 1,000 mg/kg orally administered mice as compared to OVA control mice during the experimental periods. In contrast, significant decreases of body weights were observed from the 8th treatment day with DEXA 3 mg/kg as compared to those of intact vehicle control and also from the 15th treatment day as compared to those of OVA control mice (arrows). Values are expressed as mean ± SD of 10 mice. PS = *Pelargonium sidoides*; CR = Coptis Rhizoma; PS + CR = PS and CR 2 : 1 mixed formula; OVA = ovalbumin; DEXA = dexamethasone; IP = intraperitoneal injection. ^**†**^All animals were fasted overnight. Day 0 means the day of first test substance administration/first OVA sensitization. Day 16 means the day of 24 h after last 16th test substance administration. ^a^*p* < 0.01 and ^b^*p* < 0.05 as compared with intact control by LSD test. ^c^*p* < 0.05 as compared with OVA control by LSD test.

No significant changes in the body weights or gains were detected in OVA control as compared to those of intact control throughout the experimental period. In addition, no significant changes in the body weights and gains were detected in PS + CR (300 and 1,000 mg/kg) as compared to those of OVA control during the entire experimental period. In contrast, significant (*p* < 0.01 or *p* < 0.05) decreases of body weight were observed at the 8th treatment day with DEXA as compared to those of intact control and also at the 15th treatment day as compared to those of OVA control. The body weight gains of DEXA were also significantly (*p* < 0.01 or *p* < 0.05) smaller than those of intact control and OVA control (Table 3; Figure 2).

### 3.2. Gross Inspections and Lung Weight Changes

**Figure 3:**
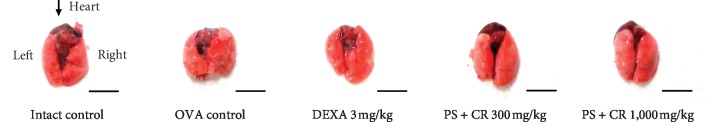
Representative lung gross images, taken from intact or OVA-induced asthmatic mice. Marked enlargement of lung-edematous changes was detected in OVA control mice as compared with intact control mice. However, these OVA-induced pulmonary edematous changes were dose-dependently inhibited by oral treatment with PS + CR 300 and 1,000 mg/kg, as being comparable to the effects of DEXA 3 mg/kg oral administration. PS = *Pelargonium sidoides*; CR = Coptis Rhizoma; PS + CR = PS and CR 2 : 1 mixed formula; OVA = ovalbumin; DEXA = dexamethasone. Scale bars = 7 mm.

**Figure 4:**
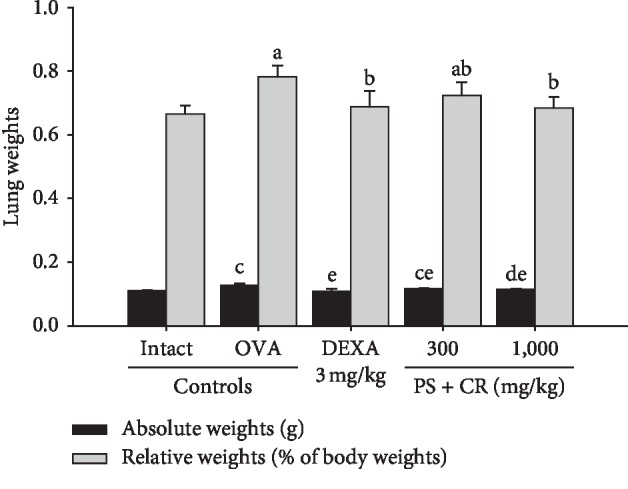
Lung weight changes in intact or OVA-induced asthmatic mice. Significant increases of lung absolute wet and relative weights were detected in OVA control mice as compared with intact control mice. However, these OVA-induced lung absolute and relative weight increases were significantly and dose-dependently inhibited by oral treatment with PS + CR 300 and 1,000 mg/kg, as being comparable to those of DEXA 3 mg/kg oral administration. Values are expressed as mean ± SD of 10 mice. PS = *Pelargonium* sidoides; CR = Coptis Rhizoma; PS + CR = PS and CR 2 : 1 mixed formula; OVA = ovalbumin; DEXA = dexamethasone. ^a^*p* < 0.01 as compared with intact control by LSD test. ^b^*p* < 0.01 as compared with OVA control by LSD test. ^c^*p* < 0.01 and ^d^*p* < 0.05 as compared with intact control by MW test. ^e^*p* < 0.01 as compared with OVA control by MW test.

Marked enlargement of lung-edematous changes and related significant (*p* < 0.01) increases of lung absolute wet and relative weights were detected in OVA control as compared with intact control. However, these OVA-induced pulmonary edematous changes and related increases of lung absolute and relative weights were significantly (*p* < 0.01) and dose-dependently inhibited by oral treatment of PS + CR as being comparable to those of DEXA (Figures 3 and 4).

The absolute lung weights in OVA control were changed by 16.82% as compared with intact control, but they were changed by −14.87, −8.92, and −11.35% in DEXA, PS + CR 300, and PS + CR 1,000, respectively, as compared to those of OVA control.

The relative lung weights in OVA control were changed by 17.51% as compared with intact control, but they were changed by −12.01, −7.49, and −12.50% in DEXA, PS + CR 300, and PS + CR 1,000, respectively, as compared to those of OVA control.

### 3.3. BALF Cytology

**Figure 5:**
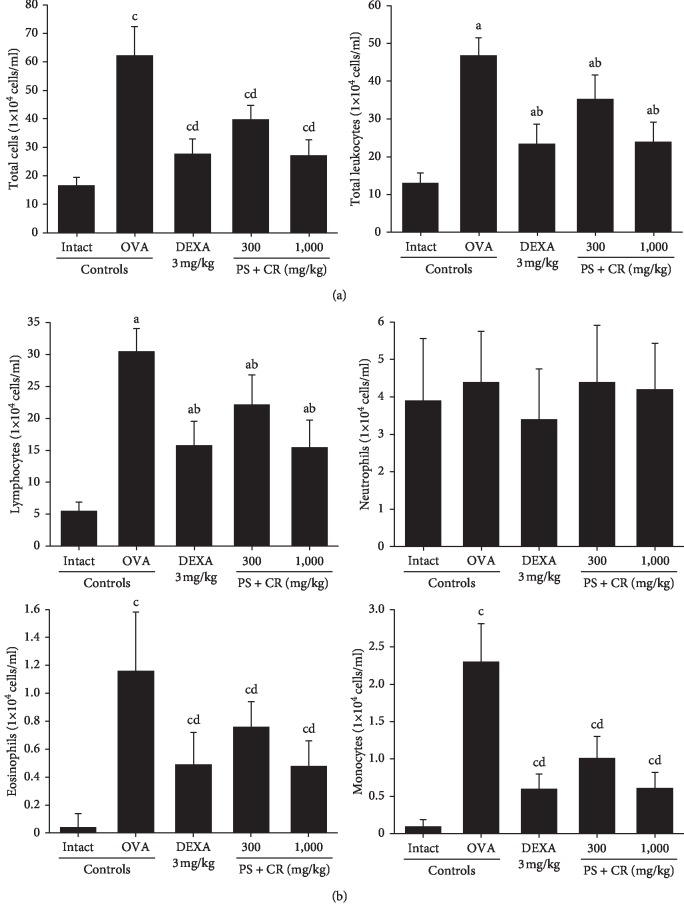
Cytology of BALF in intact or OVA-induced asthmatic mice. Significant increases of cell numbers were detected in OVA control mice as compared with intact control mice. However, these OVA-induced total cells (a) (left), total leukocytes (a) (right), lymphocytes (b) (upper left), eosinophils (b) (lower left), and monocytes (b) (lower right) were significantly and dose-dependently decreased by oral treatment with PS + CR 300 and 1,000 mg/kg, as being comparable to those of DEXA 3 mg/kg oral administration. Neutrophils of differential counts were not changed in OVA control mice as compared to those of intact control or in DEXA, PS + CR 300, and PS + CR 1,000 mg/kg as compared to those of OVA control mice (b) (upper right). Values are expressed as mean ± SD of 10 mice, ×10^4^ cells/ml. PS = *Pelargonium sidoides*; CR = Coptis Rhizoma; PS + CR = PS and CR 2 : 1 mixed formula; OVA = ovalbumin; DEXA = dexamethasone; BALF = bronchoalveolar lavage fluid. ^a^*p* < 0.01 as compared with intact control by LSD test. ^b^*p* < 0.01 as compared with OVA control by LSD test. ^c^*p* < 0.01 as compared with intact control by MW test. ^d^*p* < 0.01 as compared with OVA control by MW test.

Significant (*p* < 0.01) increases of BALF total cells (Figure 5(a), left), total leukocytes (Figure 5(a), right), lymphocytes (Figure 5(b), upper left), eosinophils (Figure 5(b), lower left), and monocytes (Figure 5(b), lower right) were detected in OVA control as compared with intact control. However, these OVA-induced increases were significantly (*p* < 0.01) and dose-dependently inhibited by PS + CR. However, significant changes on the BALF neutrophil numbers were not demonstrated in OVA control mice as compared to those of intact control or in DEXA, PS + CR 300, and PS + CR 1,000 as compared to those of OVA control mice (Figure 5(b), upper right).

The BALF total cell numbers in OVA control were changed by 272.46% as compared with intact control but were changed by −55.31, −36.01, and −56.27% in DEXA, PS + CR 300, and PS + CR 1,000 as compared to those of OVA control, and the total leukocyte numbers in OVA control were changed by 260.00% as compared with intact control but were changed by −50.00, −24.57, and −48.72% in DEXA, PS + CR 300, and PS + CR 1,000, respectively, as compared to those of OVA control.

In the BALF, the lymphocyte numbers in OVA control were changed by 454.55% as compared with intact control but were changed by −48.20, −27.21, and −49.18% in DEXA, PS + CR 300, and PS + CR 1,000, respectively, and the neutrophil numbers in OVA control were changed by 12.82% as compared with intact control and were changed by −22.73, 0.00, and −4.55% in DEXA, PS + CR 300, and PS + CR 1,000, respectively. The eosinophil numbers in OVA control mice were changed by 2800.00% as compared with intact control but were changed by −57.76, −34.48, and −58.62% in DEXA, PS + CR 300, and PS + CR 1,000, respectively, and the monocyte numbers in OVA control were changed by 2200.00% as compared with intact control but were changed by −73.91, −56.09, and −73.48% DEXA, PS + CR 300, and PS + CR 1,000, respectively.

### 3.4. Serum OVA-sIg E Levels

**Figure 6:**
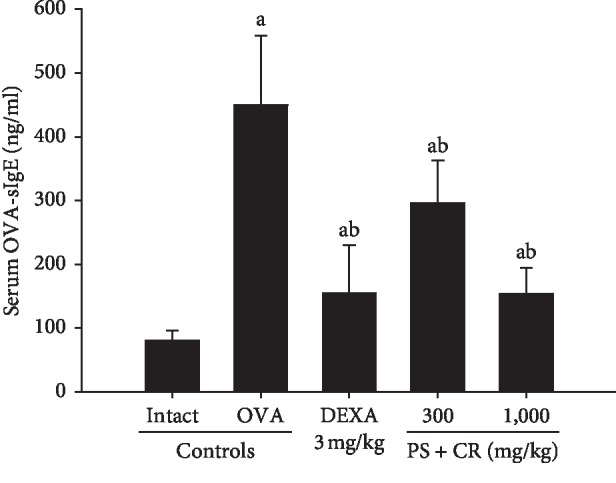
Serum OVA-sIgE level changes in intact or OVA-induced asthmatic mice. Significant increases of serum OVA-sIgE levels were detected in OVA control mice as compared with intact control mice. However, these OVA-induced increases of serum OVA-sIgE levels were significantly and dose-dependently inhibited by oral treatment with PS + CR 300 and 1,000 mg/kg, as being comparable to those of DEXA 3 mg/kg oral administration. Values are expressed as mean ± SD of 10 mice. PS = *Pelargonium sidoides*; CR = Coptis Rhizoma; PS + CR = PS and CR 2 : 1 mixed formula; OVA = ovalbumin; DEXA = dexamethasone; sIg = specific immunoglobulin. ^a^*p* < 0.01 as compared with intact control by MW test. ^b^*p* < 0.01 as compared with OVA control by MW test.

Significant (*p* < 0.01) increases of serum OVA-sIgE levels were detected in OVA control mice as compared with intact control. However, these OVA-induced increases of serum OVA-sIgE levels were significantly (*p* < 0.01) and dose-dependently inhibited by oral treatment of PS + CR (300 and 1,000 mg/kg) as compared to those of DEXA (3 mg/kg) (Figure 6).

The serum OVA-sIgE levels in OVA control mice were changed by 457.25% as compared with intact control but were changed by −65.64, −34.11, and −65.87% in DEXA, PS + CR 300, and PS + CR 1,000, respectively.

### 3.5. BALF IL-4 and IL-5 Contents

**Figure 7:**
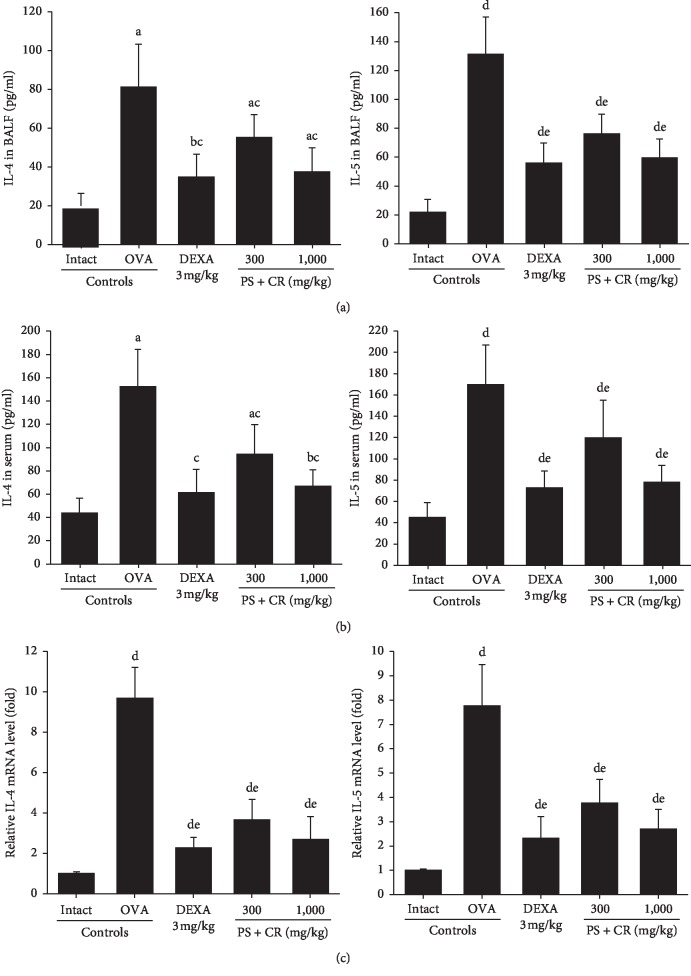
Cytokine levels in the BALF and lung with mRNA expressions in lungs. Significant increases of IL-4 and IL-5 contents were detected in OVA control mice as compared with intact control mice. However, these OVA-induced IL-4 and IL-5 contents in BALF (a), serum (b), and lung tissue (c) were significantly and dose-dependently decreased by oral treatment with PS + CR 300 and 1,000 mg/kg, as being comparable to those of DEXA 3 mg/kg oral administration. Values are expressed as mean ± SD of 10 mice. PS *=* *Pelargonium sidoides*; CR = Coptis Rhizoma; PS + CR = PS and CR 2 : 1 mixed formula; OVA = ovalbumin; DEXA = dexamethasone; BALF = bronchoalveolar lavage fluid; IL = interleukin; GAPDH = glyceraldehyde 3-phosphate dehydrogenase. ^a^*p* < 0.01 and ^b^*p* < 0.05 as compared with intact control by LSD test. ^c^*p* < 0.01 as compared with OVA control by LSD test. ^d^*p* < 0.01 as compared with intact control by MW test. ^e^*p* < 0.01 as compared with OVA control by MW test.

Significant (*p* < 0.01) increases of BALF IL-4 and IL-5 contents were detected in OVA control mice as compared with intact control. However, these OVA-induced cytokine elevations in the BALF were significantly (*p* < 0.01) and dose-dependently inhibited by oral treatment of PS + CR (300 and 1,000 mg/kg), as compared to those of DEXA (3 mg/kg), in the present experiment (Figure 7(a)).

The BALF IL-4 contents in OVA control were changed by 309.55% as compared with intact control but were changed as –57.18, −32.02, and −53.74% in DEXA, PS + CR 300, and PS + CR 1,000 as compared to those of OVA control. The BALF IL-5 contents in OVA control were changed by 495.93% as compared with intact control but were changed by −57.18, −41.99, and −54.59% in DEXA, PS + CR 300, and PS + CR 1,000, respectively, as compared to those of OVA control.

### 3.6. Serum IL-4 and IL-5 Levels

Significant (*p* < 0.01) increases of serum IL-4 and IL-5 levels were detected in OVA control as compared with intact control. However, these OVA-induced cytokine elevations in the serum were significantly (*p* < 0.01) and dose-dependently inhibited by oral treatment of PS + CR (300 and 1,000 mg/kg) as comparable to those of DEXA (3 mg/kg) in this experiment (Figure 7(b)).

The serum IL-4 levels in OVA control mice were changed by 246.03% as compared with intact control, but they were changed by −59.44, −37.94, and −56.03% in DEXA, PS + CR 300, and PS + CR 1,000, respectively, as compared to those of OVA control mice. The serum IL-5 levels in OVA control mice were changed by 273.63% as compared with intact control but were changed by −56.94, −29.29, and −53.94% in DEXA, PS + CR 300, and PS + CR 1,000, respectively, as compared to those of OVA control.

### 3.7. Lung IL-4 and IL-5 mRNA Expressions

Significant (*p* < 0.01) increases of lung tissue IL-4 and IL-5 mRNA expressions were detected in OVA control as compared with intact control. However, these OVA-induced over expressions of IL-4 and IL-5 in the lung tissue were significantly (*p* < 0.01) and dose-dependently inhibited by oral treatment of PS + CR (300 and 1,000 mg/kg) as compared to those of DEXA (3 mg/kg) in this experiment (Figure 7(c)).

The lung tissue IL-4 mRNA expressions in OVA control were changed by 842.72% as compared with intact control, but they were changed by −76.31, −62.10, and −71.99% in DEXA, PS + CR 300, and PS + CR 1,000, respectively, as compared to those of OVA control. The lung tissue IL-5 mRNA expressions in OVA control were changed by 671.29% as compared with intact control but were changed by −69.96, −51.35, and −65.08% in DEXA, PS + CR 300, and PS + CR 1,000, respectively, as compared to those of OVA control.

### 3.8. Lung Histopathology

**Figure 8:**
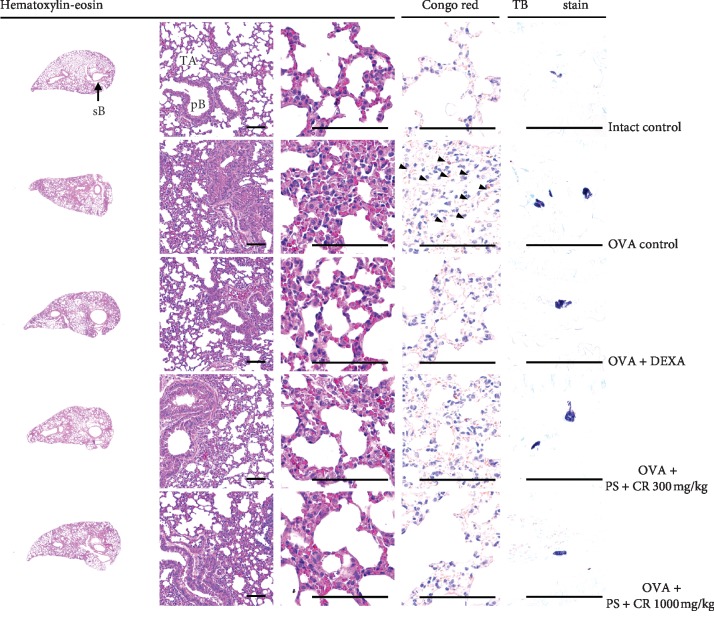
Representative lung histological images, taken from intact or OVA-induced asthmatic mice. Noticeable sarcomatous asthmatic changes, hyperplasia of mucous producing cells in bronchioles, thickening of alveolar septum, and infiltration of inflammatory cells, mast cells, and eosinophils, were observed in OVA control mice lung tissues as compared to those of intact control mice. However, these OVA-induced sarcomatous asthmatic histological changes were significantly and dose-dependently inhibited by oral treatment with PS + CR 300 and 1,000 mg/kg, as comparable to the effects of DEXA 3 mg/kg oral administration. PS = *Pelargonium sidoides*; CR = Coptis Rhizoma; PS + CR = PS and CR 2 : 1 mixed formula; OVA = ovalbumin; DEXA = dexamethasone; sB = secondary bronchus; pB = primary bronchiole; TA = terminal respiratory bronchiole-alveoli. Scale bars = 200 *μ*m.

**Figure 9:**
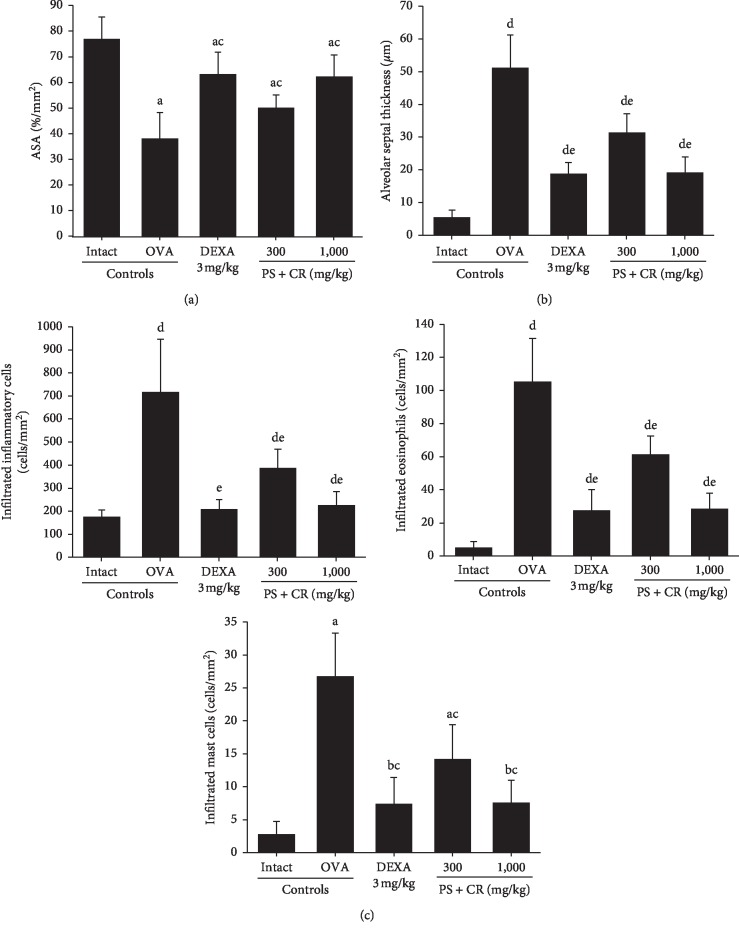
Histomorphometrical analysis of lung tissues, taken from intact or OVA-induced asthmatic mice. Significant decrease of the mean ASA was detected in OVA control mice as compared with intact control mice. However, the mean ASA of OVA-induced asthmatic mice (a) was significantly increased by oral treatment with PS + CR 300 and 1,000 mg/kg, as being comparable to that of DEXA 3 mg/kg oral administration. Significant increases of the mean alveolar septal thicknesses, infiltrated inflammatory cells, mast cells, and eosinophils around the alveolar regions were detected in OVA control mice as compared with intact control mice. However, the mean alveolar septal thicknesses (b), infiltrated inflammatory cells (c) (upper left), mast cells (c) (lower left), and eosinophils (c) (upper right) of OVA-induced asthmatic mice were significantly and dose-dependently decreased by oral treatment with PS + CR 300 and 1,000 mg/kg, as being comparable to those of DEXA 3 mg/kg oral administration. Values are expressed as mean ± SD of 10 mice. PS = *Pelargonium sidoides*; CR = Coptis Rhizoma; PS + CR = PS and CR 2 : 1 mixed formula; OVA = ovalbumin; DEXA = dexamethasone; ASA = alveolar surface area. ^a^*p* < 0.01 and ^*b*^*p* < 0.05 as compared with intact control by LSD test. ^c^*p* < 0.01 as compared with OVA control by LSD test. ^d^*p* < 0.01 as compared with intact control by MW test. ^e^*p* < 0.01 as compared with OVA control by MW test.

Noticeable sarcomatous asthmatic changes, hyperplasia of mucous producing cells in bronchioles, thickening of alveolar septum, and infiltration of inflammatory cells, mast cells, and eosinophils, were demonstrated in OVA control mice lung tissues as compared to those of intact control mice. These were reconfirmed by histomorphometrical analysis. There were significant (*p* < 0.01) increases of the mean alveolar septal thicknesses, infiltrated inflammatory cells, mast cells, and eosinophil numbers around the alveolar regions, and there was a decrease of mean ASA in OVA control as compared to intact control. However, these OVA-induced sarcomatous asthmatic histological changes were significantly (*p* < 0.01) and dose-dependently inhibited by oral treatment of PS + CR (300 and 1,000 mg/kg), constantly as comparable to that by DEXA (3 mg/kg) in this experiment (Figures 8 and 9).

The mean ASA in OVA control were changed by −50.43% as compared with intact control but were changed by 65.62, 31.58, and 63.15% in DEXA, PS + CR 300, and PS + CR 1,000, respectively, as compared to those of OVA control.

The mean alveolar septal thicknesses in OVA control were changed by 822.16% as compared with intact control but were changed by −63.25, −38.65, and −62.37% in DEXA, PS + CR 300, and PS + CR 1,000, respectively, as compared to those of OVA control mice.

The numbers of inflammatory cells infiltrated in alveolar regions of OVA control were changed by 306.47% as compared with intact control but were changed by −70.79, −45.80, and −68.57% in DEXA, PS + CR 300, and PS + CR 1,000, respectively, as compared to those of OVA control.

The numbers of eosinophils infiltrated into alveolar regions of OVA control were changed by 1926.92% as compared with intact control but were changed as −73.81, −41.75, and −72.87% in DEXA, PS + CR 300, and PS + CR 1,000, respectively, as compared to those of OVA control. The numbers of mast cells infiltrated into alveolar regions of OVA control were changed by 857.14% as compared with intact control but were changed by −72.39, −47.01, and −71.64% in DEXA, PS + CR 300, and PS + CR 1,000, respectively, as compared to those of OVA control.

## 4. Discussion

In South Africa, the root of the PS is traditionally used to treat respiratory and gastrointestinal infections, dysmenorrhea, and hepatic disorders [33, 34]. It has also been used in tuberculosis, and its medicinal properties include antibacterial, antifungal, antiviral, and immune modulatory activities [13]. On the other hand, CR has been used to treat gastroenteric disorders, cardiovascular diseases, fever, cancer, and liver injuries [35, 36]. Recent research has shown that the root has pharmacological properties, such as antioxidant, anticancer, and anti-inflammatory activities [17, 18]. In particular, we previously reported that PS + CR has potent anti-inflammatory activities, *in vitro* and *in vivo* [19].

Here, we observed the possible beneficial antiasthma effects of PS + CR on the OVA-induced asthma C57BL/6J mice. This finding gives us evidence that oral treatment with PS + CR showed favorable effects on OVA-induced asthma in C57BL/6J mice, through anti-inflammatory and antiallergic activities. Therefore, PS + CR have enough potential as a new therapeutic agent or as an ingredient of medicinal food for various allergic and inflammatory respiratory diseases including asthma.

OVA-induced asthma model is a representative asthma animal model that resembles human asthma [20, 21]. An OVA-evoked asthma model using C57BL/6J mice is useful to evaluate potential therapeutic candidates for asthma, because several parameters are clearly increased by OVA if evoked within three days after challenge. An OVA-evoked asthmatic C57BL/6J mouse model is generally used to detect the efficacy of the test articles on allergic asthma [20, 21]. As a result of OVA sensitization and boosting, significant increases of lung weights, total and differential cell counts of leukocytes in BALF, serum OVA-sIgE levels, IL-4 and IL-5 levels in BALF and lung tissue homogenate, and IL-4 and IL-5 mRNA levels in lung tissue homogenates were observed in OVA control as compared to those of intact control. In addition, OVA control showed a significant decrease of mean ASA and increases of alveolar septal thickness, numbers of inflammatory cells, mast cells, and eosinophils infiltrated into alveolar regions as compared to intact control.

Effects of PS + CR were evaluated in lung weight, histomorphometrical changes of airway systems with cytokine changes, and cellular components (recruitment of eosinophils and neutrophils) of BALF, lung, and/or peripheral blood [20, 21]. Moreover, oral treatment with PS + CR (300 and 1,000 mg/kg) inhibited the classic OVA-induced allergic and inflammatory asthmatic changes in a dose-dependent manner, which is similar to previous findings [37, 38]. *Erythronium japonicum* reduced the number of WBC and the IgE level induced by OVA in the bronchoalveolar fluid, and *Perillae Fructus* suppressed lung weight, the number of inflammatory cells in the lung, and BALF [37, 38].

The effect of PS + CR was compared with those of DEXA. It is well clarified that DEXA is a glucocorticoid that is widely used as an anti-inflammatory control drug for developing new antiasthmatic agents [20, 22], as potent alternative antiasthmatic natural agents or functional foods. Like other side effects commonly known in previous studies [20, 39], significant decreases of body weights were observed from the 8th treatment day with DEXA 3 mg/kg as compared to those of intact control and from the 15th treatment day as compared to those of OVA control mice in this study. In addition, the body weight gains during the 16 days of the experimental period were also significantly decreased in mice treated with DEXA 3 mg/kg and were less than those of intact control and OVA control mice, respectively. However, no significant changes in the body weights or gains were detected in PS + CR 300 or 1,000 as compared to those of OVA control mice throughout the experimental period, suggesting fewer side effects. Therefore, more detailed mechanical studies (i.e., transforming growth factors and mitogen-activated protein kinases) and more plentiful *in vivo* studies should be tested in future [40, 41].

Challenge with OVA induced significant airways architectural and physiological changes [27]. Biochemically, lung weight index, that is, relative weight to body weight, is considered a good marker of vascular permeability and еdеma [42]. Mice challenged with OVA showed elevated microvascular leakage, resulting in increased lung еdеma and related increases of lung weights [43, 44]. In this study, marked enlargement of lung-edematous changes and related significant increases of lung absolute wet and relative weights were detected in OVA, which are inhibited by oral treatment with PS + CR (300 and 1,000 mg/kg). These findings are clear evidence that PS + CR showed the inhibitory effects on OVA-induced lung edema and related increases of lung index, through reduction of microvascular leakage mediated by anti-inflammatory activity, as being comparable to those of DEXA (3 mg/kg).

OVA challenge led to a significant elevation in the number of total white blood cells, lymphocytes, monocytes, and eosinophils in BALF, confirming the previous study in which OVA induced significant elevation of cell counts in BALF mice [20, 27]. The current results showed that OVA-induced increases of BALF total cells, total leukocytes, lymphocytes, eosinophils, and monocytes were significantly and dose-dependently inhibited by oral treatment with PS + CR (300 and 1,000 mg/kg), as being comparable to the effects of DEXA (3 mg/kg), as anti-inflammatory activity that mediates antiasthmatic effects.

Allergic asthma is a chronic airway inflammation disease, and high serum levels of IgE, persistent airway hyperresponsiveness, and goblet-cell hyperplasia are observed [3]. In animal models, OVA challenges induced a significant increase in the total serum IgE and BALF IgE [3, 20, 45]. Our data showed that the serum concentration of OVA-sIgE was significantly reduced in allergic mice after treatment with PS + CR (300 and 1,000 mg/kg), as is comparable to the effects of DEXA (3 mg/kg) administration. This result suggests that PS + CR affects the allergic asthma that developed in an IgE-dependent manner, as being comparable to DEXA (3 mg/kg) at an oral dose level of 1,000 mg/kg.

Th2 cytokines play an essential role in the pathogenesis of allergic airway inflammation [3, 46]. IL-4 has important roles in allergic inflammation and airway remodeling [8]. IL-5 is the most specific to eosinophils and is responsible for eosinophil growth, differentiation, mobilization, recruitment, activation, and survival [10, 47]. In the present ELISA and quantitative RT-PCR analysis, significant increases of BALF and lung IL-4 and IL-5 contents were detected in OVA control as compared to intact control. In addition, upregulation of IL-4 and IL-5 mRNA in lung tissue homogenates was also demonstrated in OVA control as compared to intact control. However, such OVA-induced upregulation of IL-4 and IL-5 mRNA expressions and related elevations in the BALF and lungs were significantly and dose-dependently inhibited by oral treatment with PS + CR (300 and 1,000 mg/kg), as being comparable to the results of DEXA (3 mg/kg) administration, at least in this study. Therefore, the inhibition of asthmatic symptoms by PS + CR may be associated with the reduction of IL-4 and IL-5 production and the eosinophilia aggregation into the lungs, as comparable to DEXA (3 mg/kg), at oral dose level of 1,000 mg/kg, once again.

The main pathological changes that highlight asthma are mucus overproduction, airway obstruction, and increased infiltration of inflammatory cells, including mast cells, eosinophils, and lymphocytes [48]. Histopathologically, marked sarcomatous changes, hyperplasia of mucous producing cells in bronchioles, thickening of alveolar septum, and infiltration of inflammatory cells, mast cells, and eosinophils, were observed in OVA-challenged mouse lung tissues [20, 21, 27] and also in our OVA control mice. The ASA (%/mm^2^) reflected the pulmonary functions, gas exchange capacities, and decreases of ASA indicated a reduction of the lung gas exchange surface, that is, the decreases of lung capacities in various lung diseases, including asthma [20, 28]. Our data showed that the histopathological sarcomatous changes were significantly reduced in allergic mice after PS + CR 300 and 1,000 mg/kg oral administration, constantly as comparable to those of DEXA (3 mg/kg) administration. These findings are direct evidence that PS + CR (300 and 1,000 mg/kg) showed dose-dependent favorable inhibitory effects on OVA-induced asthma in C57BL/6J mice, perhaps through anti-inflammatory and antiallergic activities, as comparable to those of DEXA (3 mg/kg) administration.

## 5. Conclusion

In the present analysis, significant increases of lung weights, total and differential cell counts of leukocytes in BALF, serum OVA-sIgE levels, and IL-4 and IL-5 levels were observed in OVA control as compared to those of intact vehicle control. In addition, OVA control showed significant decreases of mean ASA and increases of alveolar septal thickness and numbers of inflammatory cell, mast cell, and eosinophils infiltrated into alveolar regions as compared to those of intact control. These results represent classic OVA-induced allergic and inflammatory asthmatic changes. However, these OVA-induced allergic and inflammatory asthmatic changes were significantly inhibited by oral treatment with PS + CR in a dose-dependent manner, as compared to DEXA (3 mg/kg). These findings are definitive evidence that PS + CR showed dose-dependent favorable effects on OVA-induced asthma in C57BL/6J mice, through anti-inflammatory and antiallergic activities. Therefore, PS + CR have potential as a new therapeutic agent or ingredient of medicinal food for various allergic and inflammatory respiratory diseases, including asthma. However, more detailed mechanical studies and more plentiful *in vivo* studies should be tested in future.

## Figures and Tables

**Table 1:** Actual compositions of test substances used in this study.

Groups	Compositions (mg)			Vehicle (10 ml)
	DEXA	PS	CR	
Controls				
Intact	0	0	0	0.5% CMC
OVA	0	0	0	0.5% CMC
Reference				
DEXA	3 (45.56)	0	0	Saline
PS + CR				
300 mg/kg	0	200	100	0.5% CMC
1,000 mg/kg	0	666.67	333.33	0.5% CMC

PS = *Pelargonium sidoides*; CR = Coptis Rhizoma; PS + CR = PS and CR 2 : 1 mixed formula; OVA = ovalbumin; CMC = carboxymethyl cellulose; DEXA = dexamethasone.

**Table 2:** Oligonucleotides for quantitative RT-PCR used in this study.

Target	5'–3′	Sequence
Interleukin-4	Forward	GAATGTACCAGGAGCCATATC
	Reverse	CTCAGTACTACGAGTAATCCA
Interleukin-5	Forward	TCACCGAGCTCTGTTGACAA
	Reverse	CCACACTTCTCTTTTTGGCG
GAPDH	Forward	CATCTTCCAGGAGCGAGACC
	Reverse	AAGCCATGCCAATGTTGTCT

RT-PCR = reverse transcription polymerase chain reaction; GAPDH = glyceraldehyde 3-phosphate dehydrogenase.

**Table 3:** Body weight gains in intact or OVA-induced asthmatic mice.

Groups	Periods			
	Body weights (g)			Weight gains [B-A]
	Day 1	Day 0 (a)^*∗*^	Day 16 (B)^*∗*^	
Controls				
Intact	18.16 ± 0.56	14.85 ± 0.50	16.45 ± 0.46	1.60 ± 0.30
OVA	18.18 ± 0.46	14.92 ± 0.51	16.36 ± 0.71	1.44 ± 0.43
DEXA 3 mg/kg	18.20 ± 0.64	15.01 ± 0.53	15.84 ± 0.73	0.83 ± 0.44^ab^
PS + CR				
300 mg/kg	18.18 ± 0.80	14.88 ± 0.53	16.12 ± 0.74	1.24 ± 0.45
1,000 mg/kg	18.16 ± 0.58	15.08 ± 0.49	16.59 ± 0.84	1.51 ± 0.67

PS = *Pelargonium sidoides*; CR = Coptis Rhizoma; PS + CR = PS and CR 2 : 1 mixed formula; OVA = ovalbumin; CMC = carboxymethyl cellulose; DEXA = dexamethasone. Day 16 means the day of 24 h after last 16th PS + CR or DEXA administration ^a^*p* < 0.01 as compared with intact control by LSD test. ^b^*p* < 0.01 as compared with OVA control by LSD test.

## Data Availability

The data used to support the findings of this study are included within the article.

## References

[1] Takeda K., Thurman J. M., Tomlinson S. (2012). The critical role of complement alternative pathway regulator factor H in allergen-induced airway hyperresponsiveness and inflammation. *The Journal of Immunology*.

[2] Holgate S. T. (2008). The airway epithelium is central to the pathogenesis of asthma. *Allergology International*.

[3] Chu X., Wei M., Yang X. (2012). Effects of an anthraquinone derivative from rheum officinale baill, emodin, on airway responses in a murine model of asthma. *Food and Chemical Toxicology*.

[4] Inam A., Shahzad M., Shabbir A., Shahid H., Shahid K., Javeed A. (2017). Carica papaya ameliorates allergic asthma via down regulation of IL-4, IL-5, eotaxin, TNF-*α*, NF-*ĸ*B, and iNOS levels. *Phytomedicine*.

[5] Liu X., Yu D., Wang T. (2016). Sappanone A attenuates allergic airway inflammation in ovalbumin-induced asthma. *International Archives of Allergy and Immunology*.

[6] Liang P., Peng S., Zhang M., Ma Y., Zhen X., Li H. (2017). Huai Qi Huang corrects the balance of Th1/Th2 and Treg/Th17 in an ovalbumin-induced asthma mouse model. *Bioscience Reports*.

[7] Venturini C. L., Macho A., Arunachalam K. (2018). Vitexin inhibits inflammation in murine ovalbumin-induced allergic asthma. *Biomedicine & Pharmacotherapy*.

[8] Jie Z., Jin M., Cai Y. (2009). The effects of Th2 cytokines on the expression of ADAM33 in allergen-induced chronic airway inflammation. *Respiratory Physiology & Neurobiology*.

[9] Deo S., Mistry K., Kakade A., Niphadkar P. (2010). Role played by Th2 type cytokines in IgE mediated allergy and asthma. *Lung India*.

[10] Simon D., Braathen L. R., Simon H.-U. (2004). Eosinophils and atopic dermatitis. *Allergy*.

[11] Cameron L., Christodoulopoulos P., Lavigne F. (2000). Evidence for local eosinophil differentiation within allergic nasal mucosa: inhibition with soluble IL-5 receptor. *The Journal of Immunology*.

[12] Rana S., Shahzad M., Shabbir A. (2016). Pistacia integerrima ameliorates airway inflammation by attenuation of TNF-*α*, IL-4, and IL-5 expression levels, and pulmonary edema by elevation of AQP1 and AQP5 expression levels in mouse model of ovalbumin-induced allergic asthma. *Phytomedicine*.

[13] Brendler T., van Wyk B.-E. (2008). A historical, scientific and commercial perspective on the medicinal use of pelargonium sidoides (*Geraniaceae*). *Journal of Ethnopharmacology*.

[14] Roth M., Fang L., Stolz D., Tamm M. (2019). Pelargonium sidoides radix extract EPs 7630 reduces rhinovirus infection through modulation of viral binding proteins on human bronchial epithelial cells. *PLoS One*.

[15] Tahan F., Yaman M. (2013). Can the Pelargonium sidoides root extract EPs 7630 prevent asthma attacks during viral infections of the upper respiratory tract in children?. *Phytomedicine*.

[16] Meng F. C., Wu Z. F., Yin Z. Q., Lin L. G., Wang R., Zhang Q. W. (2018). Coptidis rhizoma and its main bioactive components: recent advances in chemical investigation, quality evaluation and pharmacological activity. *Chinese Medicine*.

[17] Kim E., Ahn S., Rhee H.-i., Lee D.-c. (2016). Coptis chinensis Franch. extract up-regulate type I helper T-cell cytokine through MAPK activation in MOLT-4 T cell. *Journal of Ethnopharmacology*.

[18] Zhao H., Zhou S., Zhang M. (2016). An in vitro AChE inhibition assay combined with UF-HPLC-ESI-Q-TOF/MS approach for screening and characterizing of AChE inhibitors from roots of *Coptis chinensis* Franch. *Journal of Pharmaceutical and Biomedical Analysis*.

[19] Park S. M., Min B. G., Jung J. Y. (2018). Combination of *Pelargonium sidoides* and *Coptis chinensis* root inhibits nuclear factor kappa B-mediated inflammatory response in vitro and in vivo. *BMC Complementary and Alternative Medicine*.

[20] Ku S. K., Kim J. W., Cho H. R. (2012). Effect of *β*-glucan originated from Aureobasidium pullulans on asthma induced by ovalbumin in mouse. *Archives of Pharmacal Research*.

[21] André D. M., Horimoto C. M., Calixto M. C., Alexandre E. C., Antunes E. (2018). Epigallocatechin-3-gallate protects against the exacerbation of allergic eosinophilic inflammation associated with obesity in mice. *International Immunopharmacology*.

[22] Kwon O.-K., Ahn K.-S., Lee M.-Y. (2008). Inhibitory effect of kefiran on ovalbumin-induced lung inflammation in a murine model of asthma. *Archives of Pharmacal Research*.

[23] Kim D. Y., Park B. S., Hong G. U. (2011). Anti-inflammatory effects of the R2 peptide, an inhibitor of transglutaminase 2, in a mouse model of allergic asthma, induced by ovalbumin. *British Journal of Pharmacology*.

[24] Livak K. J., Schmittgen T. D. (2001). Analysis of relative gene expression data using real-time quantitative PCR and the 2−ΔΔCT method. *Methods*.

[25] Lee J. E., Kang S. J., Choi S. H., Song C. H., Lee Y. J., Ku S. K. (2015). Fermentation of green tea with 2% *Aquilariae lignum* increases the anti-diabetic activity of green tea aqueous extracts in the high fat-fed mouse. *Nutrients*.

[26] Kim C.-H., Choi Y.-S., Cheong K. A., Lee A.-Y. (2013). Mechanism underlying the effect of combined therapy using glucosamine and low-dose cyclosporine A on the development of atopic dermatitis-like skin lesions in NC/Nga mice. *International Immunopharmacology*.

[27] Abdеlaziz R. R., Еlmahdy M. K., Suddеk G. M. (2018). Flavocoxid attenuates airway inflammation in ovalbumin-induced mouse asthma model. *Chemico-Biological Interactions*.

[28] Lebargy F., Lenormand E., Pariente R., Fournier M. (1987). Morphological changes in rat tracheal mucosa immediately after antigen challenge. *Bulletin europeen de physiopathologie respiratoire*.

[29] Levene A. (1981). Pathological factors influencing excision of tumours in the head and neck. Part I. *Clinical Otolaryngology*.

[30] Ludbrook J. (1997). Update: microcomputer statistics packages. A personal view. *Clinical and Experimental Pharmacology and Physiology*.

[31] Choi J.-S., Kim J.-W., Park J. B. (2017). Blood glycemia-modulating effects of melanian snail protein hydrolysates in mice with type II diabetes. *International Journal of Molecular Medicine*.

[32] Lim J. M., Lee Y. J., Cho H. R. (2018). Extracellular polysaccharides purified from *Aureobasidium pullulans* SM-2001 (polycan) inhibit dexamethasone-induced muscle atrophy in mice. *International Journal of Molecular Medicine*.

[33] Bao Y., Gao Y., Koch E., Pan X., Jin Y., Cui X. (2015). Evaluation of pharmacodynamic activities of EPs 7630, a special extract from roots of *Pelargonium sidoides*, in animals models of cough, secretolytic activity and acute bronchitis. *Phytomedicine*.

[34] Witte K., Koch E., Volk H. D., Wolk K., Sabat R. (2015). The *Pelargonium sidoides* extract EPs 7630 drives the innate immune defense by activating selected MAP Kinase pathways in human Monocytes. *PLoS One*.

[35] Yan D., Ma B., Shi R., Wang T., Ma Y. (2015). Involvement of herb-herb interactions in the influences of radix Scutellaria and *Coptis Chinensis* on the bioavailability of the anthraquinones form *Rhei Rhizoma* in rats. *European Journal of Drug Metabolism and Pharmacokinetics*.

[36] Tan H. L., Chan K. G., Pusparajah P. (2016). Rhizoma coptidis: a potential cardiovascular protective agent. *Frontiers in Pharmacology*.

[37] Seo J.-H., Bang M.-A., Kim G., Cho S. S., Park D.-H. (2016). Erythronium japonicum attenuates histopathological lung abnormalities in a mouse model of ovalbumin-induced asthma. *International Journal of Molecular Medicine*.

[38] Yim Y.-K., Lee H., Hong K.-E. (2010). Anti-inflammatory and immune-regulatory effects of subcutaneous *Perillae fructus* extract injections on OVA-induced asthma in mice. *Evidence-Based Complementary and Alternative Medicine*.

[39] Piper S. L., Laron D., Manzano G. (2012). A comparison of lidocaine, ropivacaine and dexamethasone toxicity on bovine tenocytes in culture. *The Journal of Bone and Joint Surgery. British Volume*.

[40] Wijerathne C. U. B., Seo C.-S., Song J.-W. (2017). Isoimperatorin attenuates airway inflammation and mucus hypersecretion in an ovalbumin-induced murine model of asthma. *International Immunopharmacology*.

[41] Jeon W. Y., Shin I. S., Shin H. K., Jin S. E., Lee M. Y. (2016). Aqueous Extract of Gumiganghwal-tang, a traditional herbal medicine, reduces pulmonary fibrosis by transforming growth factor-*β*1/Smad signaling pathway in murine model of chronic asthma. *PLoS One*.

[42] Tumes D. J., Cormie J., Calvert M. G. (2007). Strain-dependent resistance to allergen-induced lung pathophysiology in mice correlates with rate of apoptosis of lung-derived eosinophils. *Journal of Leukocyte Biology*.

[43] Okada S., Kita H., George T. J., Gleich G. J., Leiferman K. M. (1997). Migration of eosinophils through basement membrane components in vitro: role of matrix metalloproteinase-9. *American Journal of Respiratory Cell and Molecular Biology*.

[44] Tillie-Leblond I., Gosset P., Le Berre R. (2007). Keratinocyte growth factor improves alterations of lung permeability and bronchial epithelium in allergic rats. *European Respiratory Journal*.

[45] Habibian R., Delirezh N., Farshid A. A. (2018). The effects of bone marrow-derived mesenchymal stem cells on ovalbumin-induced allergic asthma and cytokine responses in mice. *Iranian Journal of Basic Medical Sciences*.

[46] Medoff B. D., Thomas S. Y., Luster A. D. (2008). T cell trafficking in allergic asthma: the ins and outs. *Annual Review of Immunology*.

[47] Sanderson C. (1992). Interleukin-5, eosinophils, and disease. *Blood*.

[48] Busse W. W., Calhoun W. F., Sedgwick J. D. (1993). Mechanism of airway inflammation in asthma. *American Review of Respiratory Disease*.

